# The effect of kinin B1 receptor on chronic itching sensitization

**DOI:** 10.1186/s12990-015-0070-x

**Published:** 2015-11-14

**Authors:** Yuying Liu, Jianhua Liu, Mengran Li, Sailin Dai, Jiexian Liang, Wenjin Ji

**Affiliations:** Postgraduate Institute, Southern Medical University, Guangzhou, 510015 People’s Republic of China; Department of Anesthesiology, Guangdong General Hospital, Guangdong Academy of Medical Sciences, 96 DongChuan Road, Guangzhou, 510080 People’s Republic of China; Department of Cardiovascular Surgery, Guangdong Cardiovascular Institute, Guangdong General Hospital, Guangdong Academy of Medical Sciences, 96 DongChun Road, Guangzhou, 510080 People’s Republic of China

**Keywords:** Chronic itch, Bradykinin B1 receptor, Keratinocytes cells, HaCaT cell lines

## Abstract

**Background:**

Altered kallikrein-related peptidase activity and bradykinin are associated with skin disorders in humans and mice under chronic inflammation conditions. The bradykinin B1 receptor (B1R), also known as one of the G-protein-coupled receptor family and usually absent in intact tissues and upregulated during tissue injury, is responsible for vasodilation, capillary permeability, nociceptor sensitization, and pain; it is indispensable for physiopathological progress in chronic inflammation conditions, but its roles and effectors in the itching sensation of the allergic contact dermatitis model are poorly defined.

**Results:**

We focused on incurable itching in a diphenylcyclopropenone (DCP) chronic inflammation experimental model. Preventive treatment with the B1R antagonist R892 significantly suppressed spontaneous scratching, while the B2R selective antagonist did not. B1R expression in the skin tissues of this model was detected using a quantitative, real-time polymerase chain reaction, Western blotting, and immunohistochemistry; B1R mRNA and protein levels were increased compared with a sham-treated control group. A higher B1R IHC staining signal was observed in the keratinocytes in DCP-treated mice compared with a vehicle-treated group, so we studied the B1R function when superimposed on a protease-activated receptor 2 (PAR2) background, establishing B1R as a pivotal mediator of PAR2 function in HaCaT cell lines.

**Conclusion:**

Our data provide evidence that B1R facilitates the chronic itching sensation related to keratinocytes in a DCP-treated chronic inflammation experimental model.

## Background

Atopic dermatitis (AD) is accompanied by a disturbance of sensitization, which indicates the impact of chronic inflammation on the peripheral sensory neurons. AD patients and the animal model show increased and persistent pruritus or itching like scratching behaviors.

Recently, it was suggested that bradykinin (BK) is involved in the pathological aggravation of AD, since activation of plasma kallikrein and the secretion of skin tissue kallikrein increase in human AD patients [1]. Two kallikreins (plasma and tissue) cleave kininogens to release kinins, including BK and kallidin (Lys-BK), both of which are converted to des-Arg derivatives by carboxypeptidase N or M and become BK receptor 1 (B1R) agonists [2]. BK is a mediator of inflammation; is produced from the plasma precursor kininogen by tissue injury, anoxia, or inflammation; and is responsible for nociceptor sensitization and pain, vasodilation, and capillary permeability [3–6]. Furthermore, it is reported that BK participates in the release of some europeptides such as substance P indirectly [7]. Robust calcium transients are observed in the presence of BK in primary dorsal root ganglia (DRG) culture [8].

Whether BK participates in the chronic itch progress directly on the peripheral nerve endings or indirectly is not known. It is known, however, that B1R and BK receptor 2 (B2R) are G protein-coupled receptors that mediate kinin effects. In normal skin, B2R signaling predominates, however, and cutaneous inflammation results in enhanced B1R responses.

Our previous studies have shown that itching is induced by BK, and the activation of kinin B1 receptor mediates the alloknesis response in complete Freund’s adjuvant (CFA)-inflamed mice [9]. At the same time, in the epithelium of patients with asthma, B1R is up regulated and associated with airborne allergens that may play a role in the pathophysiology of atopic and allergic diseases [10]. Stadnicki has suggested that B1R mRNA and protein increase in specimens from inflammatory bowel disease (IBD) patients and the enhanced B1R expression is related to the pathology of IBD [11, 12]. Nevertheless, a litter study on the itching sensation reported high B1R expression, focused on chronic itching in AD. Whether the expression of B1R changes and whether B1R expression is related to the etiology and development of the itching sensation in a chronic inflammatory state remains unclear.

A recent report has indicated that treatment with diphenylcyclopropenone (DCP) often results in contact dermatitis, as well as intense itching, in both humans and mice [13, 14]. If this is the case, DCP-treated mice can be considered as a chronic inflammatory in vitro model. We focus on B1R function on itching behaviors in DCP-treated mice.

In the present study, we evaluated scratching behaviors in a mouse model of AD, especially focusing on (1) whether B1R affects itching behaviors and peripheral sensory neurons directly, and (2) its possible regulatory mechanism in up regulating B1R. For this purpose, we investigated the significance of B1R based on the behavior of the animal model and the skin tissues detected by immunohistochemistry. We then explored the possible regulatory activity of B1R by correlating B1R expression with keratinocytes dysfunction. We also tried to find out whether B1R is an independent factor in differentiating and proliferating in chronic inflammatory conditions.

## Results

### Antagonism of B1R significantly inhibited scratching in DCP-treated animals, while selective antagonist of B2R did not

**Fig. 1:**
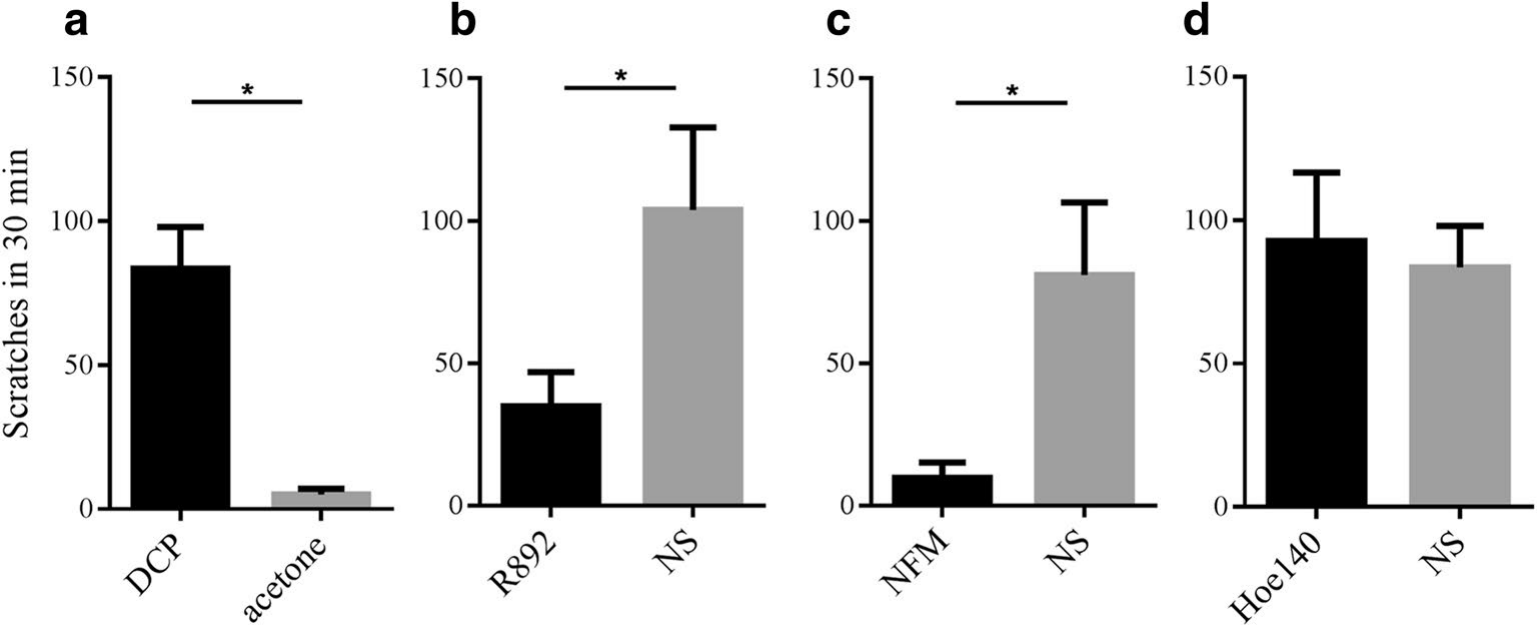
Scratching behaviors in 30 min of DCP-treated mice. Test substances were administered 30 min before observation. Graph showing increased spontaneous scratching of animal model in DCP-treated group (**a**). Preventive treatment with B1R antagonist R892 (**b**), kallikrein inhibitor NFM (**c**) significantly suppressed spontaneous scratching compared with sham-treated group (n = 8), and was significantly different from the vehicle group, while B2R antagonist HOE-140 (**d**) did not. *P < 0.05 as determined by the Student *t* test

Pruritus symptoms produced following repeated application of 1 % DCP (Aldrich Chemical Co, Milwaukee, WI, USA) was characterized by a pronounced and long-lasting increase in spontaneous scratching behavior by the hind paw (Fig. 1a). This process was observed starting on day 3, reaching a maximum on day 10 after repeated application of 1 % DCP on the seventh day (the results were not shown).

Interestingly, preventive treatment with B1R antagonist R892 (500 µg/kg, IP, 30 min before observation) (Fig. 1b), kallikrein inhibitor NFM (10 mg/kg, IP, 30 min before observation) (Fig. 1c) significantly suppressed spontaneous scratching compared with the sham-treated group. However, preventive treatment with B2R antagonist HOE-140 (300 nmol/kg, IP, 30 min before observation) during the same phase (Fig. 1d), did not significantly inhibit the scratching. Generally speaking, antagonism of B1R significantly inhibited scratching in DCP-treated animals.

Recently, a study unexpectedly reported that activation of B1R ameliorates autoimmune encephalomyelitis development [15]. For this reason, we assessed whether the activation of B1R could play a role in maintaining and promoting spontaneous, DCP-induced scratching.

### B1R is up regulated in the skin of DCP-treated mice

**Fig. 2:**
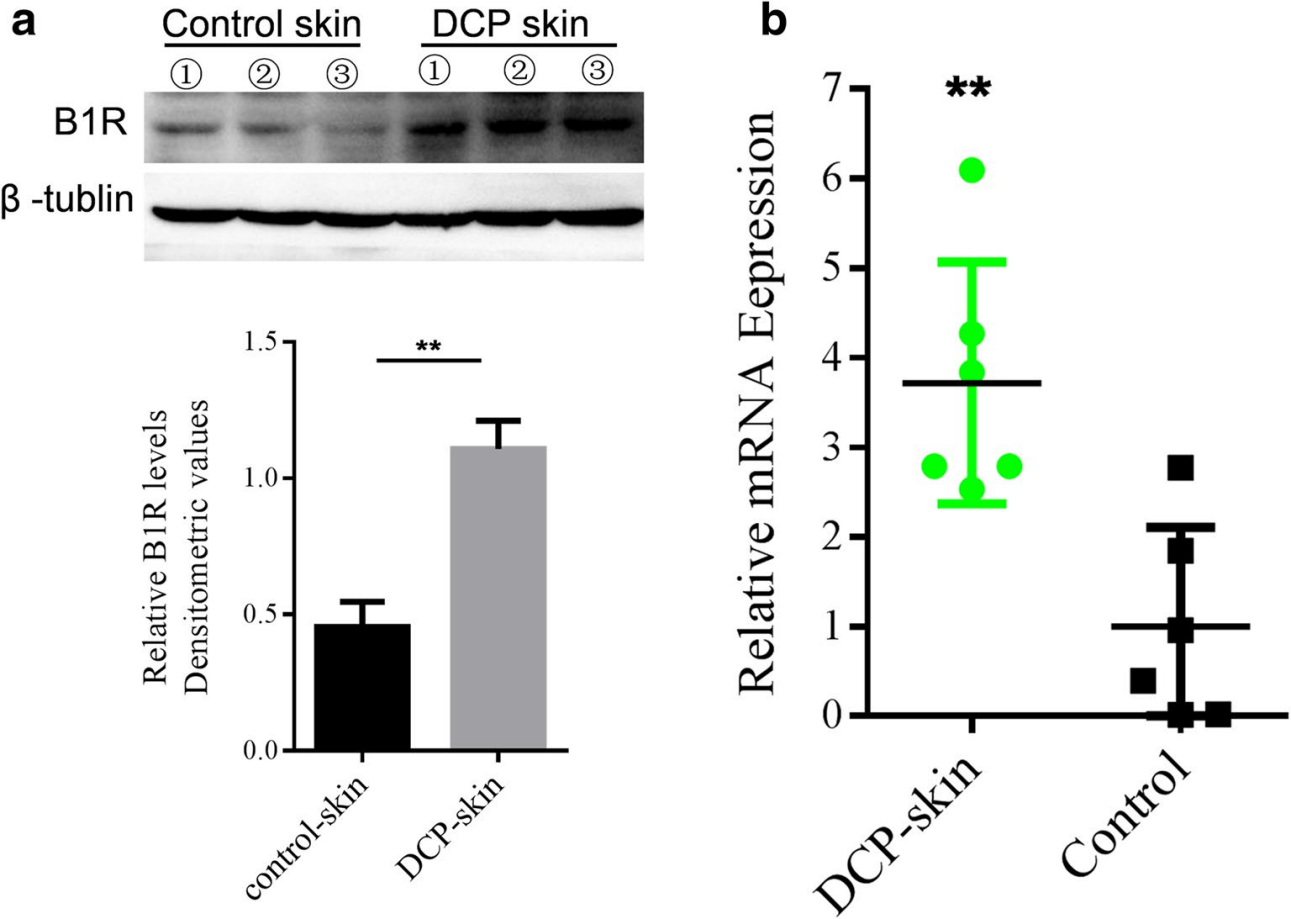
B1R is up regulated in skin tissues of DCP-treated group. Western blot (**a**) and RT-PCR (**b**) analysis of B1R in skin tissues obtained from sham (control) and DCP-treated mice. Protein expression (**a**) and PCR product (**b**) obtained with the specific B1R and β-tubulin antibodies or B1R and GAPDH primers. *Insets* (**a** *upper panels*) show a representative band of the protein expressed in both groups. Data were normalized against β-tubulin or GAPDH and expressed as the mean ± SEM of five independent mice. **P < 0.01 by the Student *t* test

To evaluate the effects of DCP on the expression of kinin B1 receptors, the mRNA and protein level from the skin of control and DCP-treated mice (on day 10 post application) were evaluated by means of real-time RT-PCR and Western blot assays, respectively. Basal expression of B1R was detected in the control group, while 10 days after DCP application, protein (Fig. 2a) and mRNA levels (Fig. 2b) of kinin B1R transcript were markedly increased in the skin tissue.

These results suggest that kinin B1 receptors exert an important role in the neuropathic hypersensitivity induced by DCP treatment.

### DCP treatment induced up regulation of B1R expression on keratinocytes, while delocalized with known neuronal maker protein gene product 9.5 (PGP9.5)

**Fig. 3:**
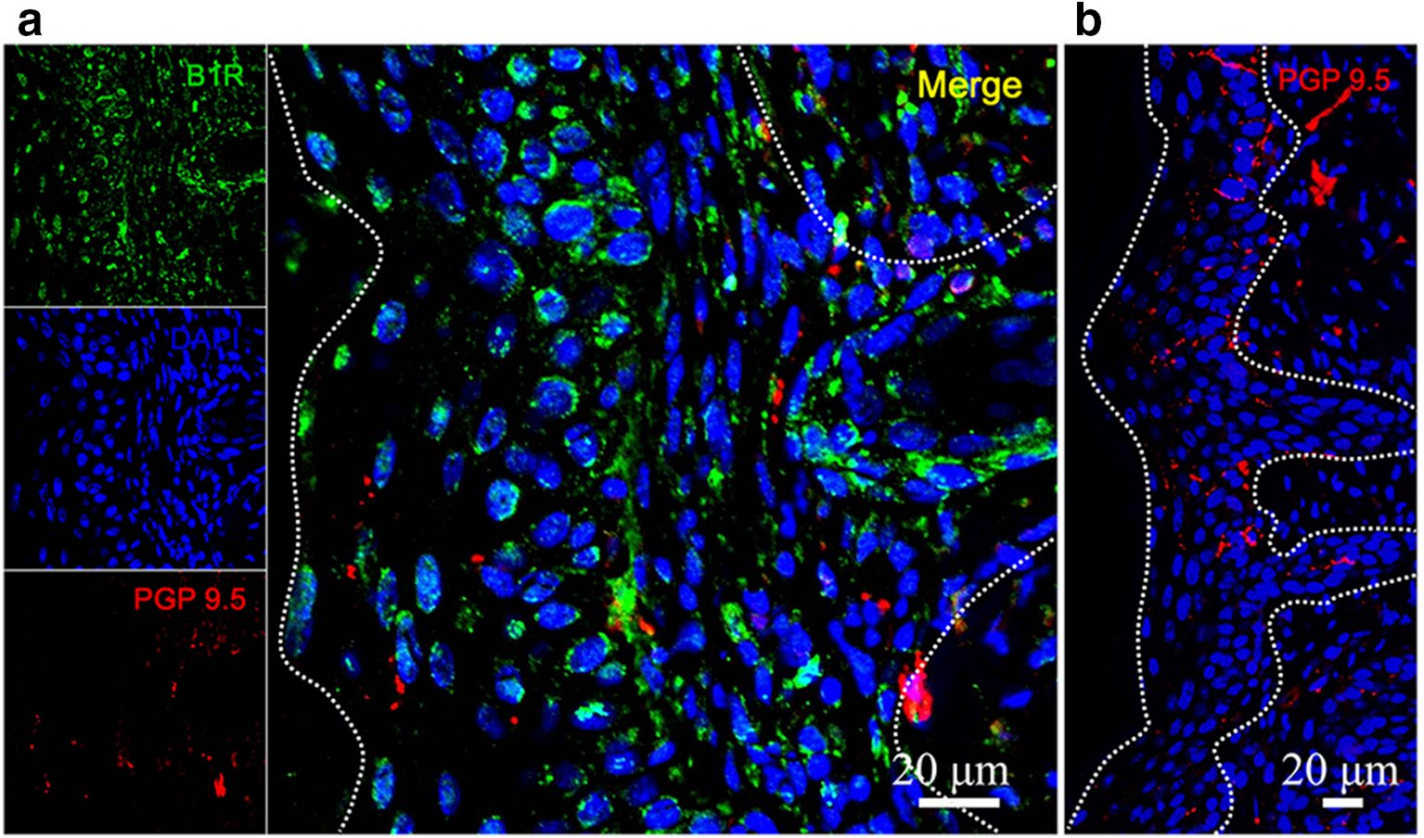
Immunofluorescence images of skin tissues in DCP-treated mice. Serial sections of post-fixed skin tissues were used (**a**) showing representative IF images with antibodies to B1R (*green*) and the neuronal marker PGP9.5 (*red*), litter level of B1R was detected in the PGP9.5 positive areas, B1R expression alone (*green*) in keratinocytes or some dermis areas (**b**). A representative IF image with antibodies to PGP9.5 was used to observe morphology of skin

**Fig. 4:**
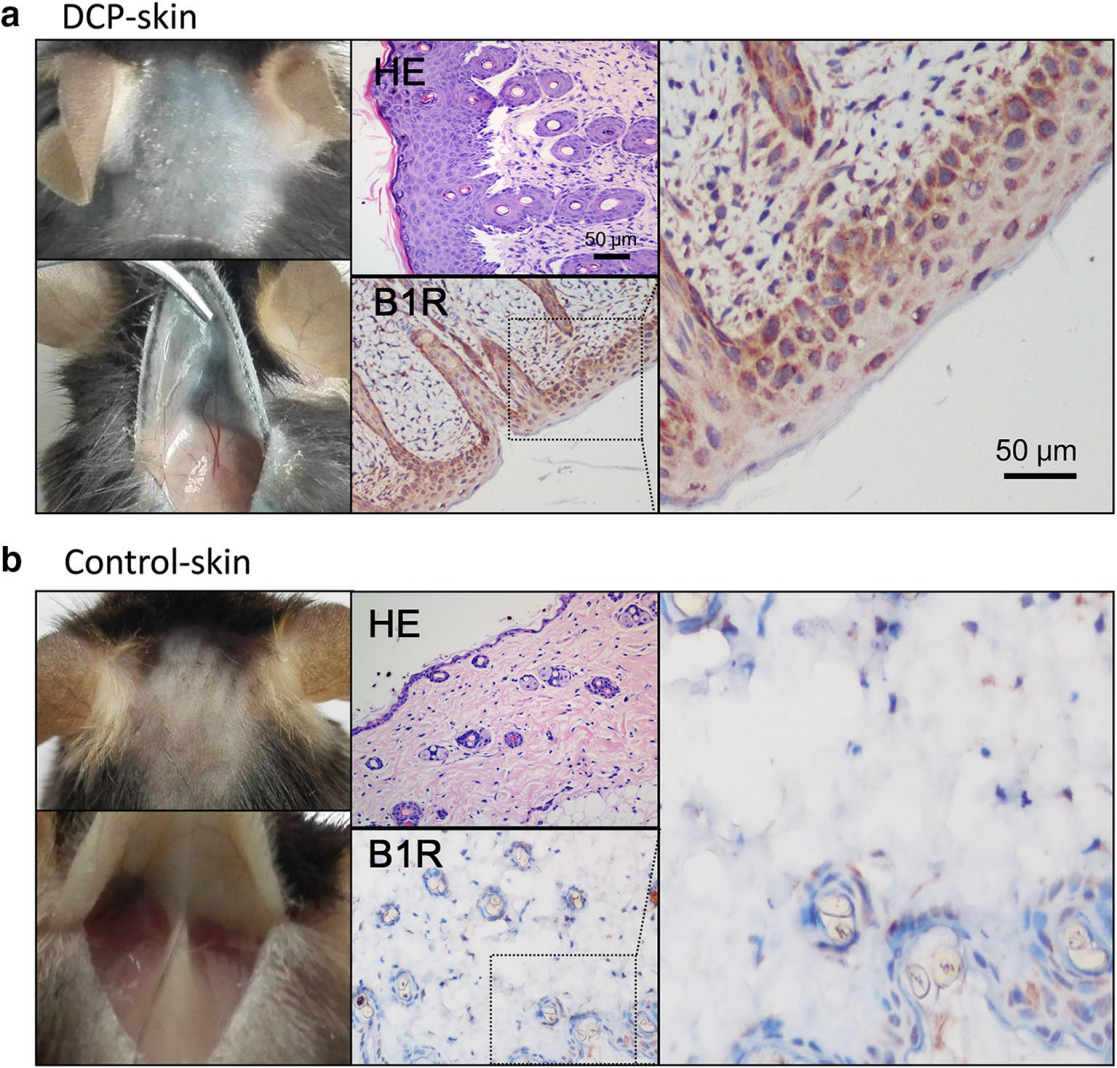
HE and IHC in mouse skin tissue. Sections of post-fixed mice skin tissues in DCP and vehicle-treated group immunostained with antibodies to B1R (**a**, **b**) showing that B1R staining signals were markedly increased in keratinocytes in DCP-treated mice. In *photos* of both groups, H&E staining was used to observe morphological and pathological changes

Previous evidence showed that thoracic spinal cord astrocytes bear the B1R in STZ-diabetic rats [16]. After acquiring all these data, we still had an unanswered question: Does the neural ending express B1R during the development of AD? For this purpose, we measured the colocalization of B1R expression with a well-known neural marker, PGP9.5, in the skin after DCP treatment. As shown in Fig. 3, little signal of B1R was detected in the PGP9.5 positive areas from treated mouse skin; nonetheless, B1R staining signal were markedly increased in keratinocytes at 10 days following DCP-treated (Fig. 4a) compared to a sham treated group (Fig. 4b). There were also other cells showing B1R signals in the dermis. These results support the concept that keratinocytes expressed and up regulated B1R expression after DCP treatment.

Thus, our data suggest that B1R primarily expressed in the keratinocytes after DCP treatment plays a relevant role in modulating scratching behaviors induced by DCP-treated in mice.

### B1R expression was induced when HaCaT cells were exposed to PAR2 agonist

Because mouse models suggest roles for B1R in keratinocytes, we hypothesized that kallikrein-related peptidase or cytokines in chronic inflammation would induce B1R production in a human keratinocyte cell line, HaCaT. A recent report has indicated that PAR-2, as a sensor for endogenous as well as exogenous proteases, plays numerous physiological and pathophysiological roles in the skin [17–19]. Sang Eun Lee reported activation of PAR2 in the lesional skin of AD led to the production of cytokines and chemokines involved in inflammation and the itching sensation, and involved PAR2/MAPKs/NF-κB signal transduction pathways [20].

In this in vitro study, we evaluated the effects of PAR2 agonist on the function of HaCaT cell lines (a keratinocyte like surface ectoderm-lineage cell line that naturally expresses PAR1 and PAR2) especially focusing on whether (1) B1R is up regulated, (2) NF-κB is involved in regulating B1R expression, and (3) B1R is involved in differentiating and proliferating keratinocytes when superimposed on a PAR2 background. For this purpose, we examined B1R regulating and the possible mechanism using a culture of HaCaT keratinocytes in the presence or absence of PAR2 agonist in a culture medium.

**Fig. 5:**
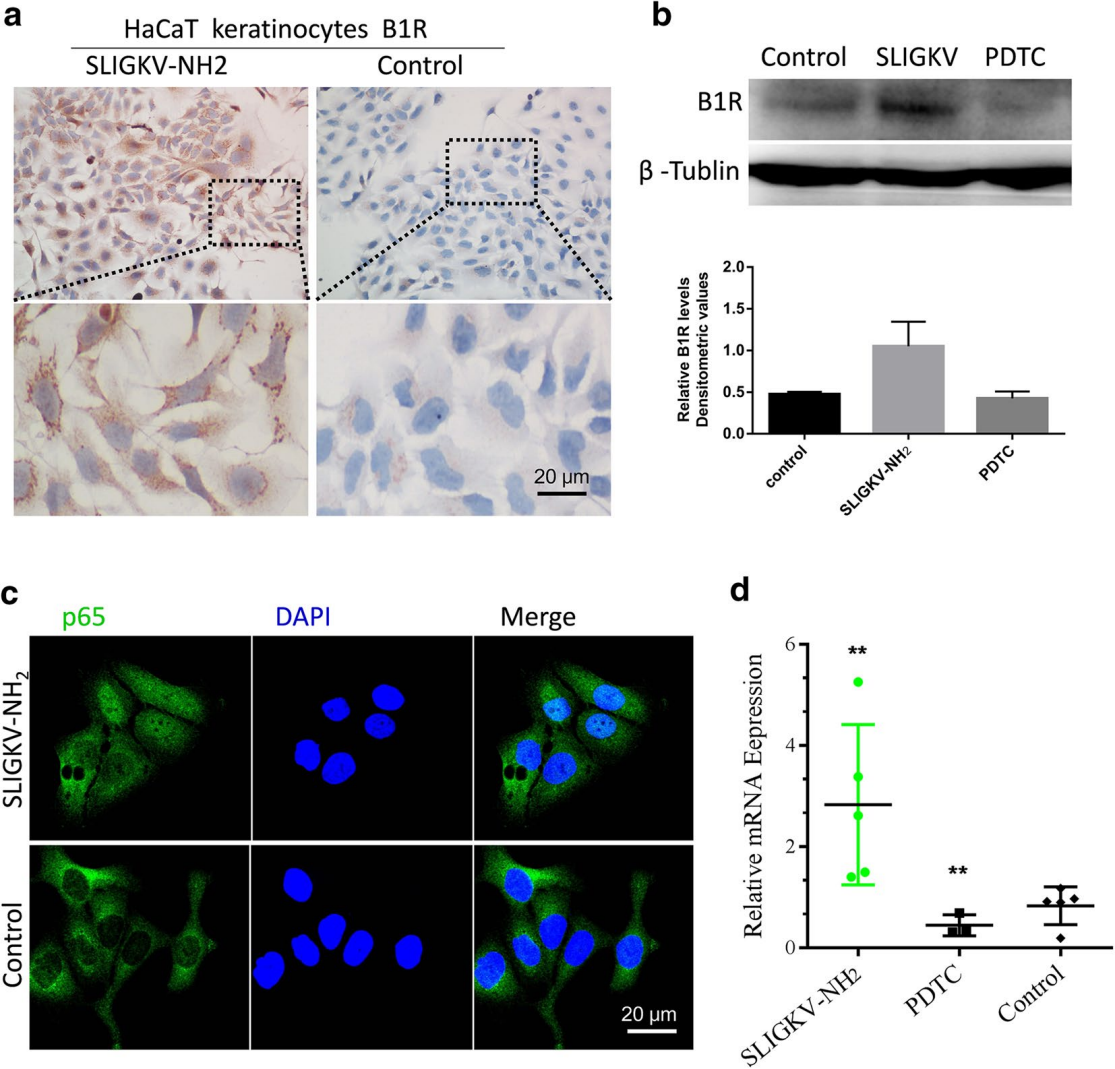
B1R expressed in HaCaT cells on a PAR2 background. Positive immunocytochemistry reaction for B1R was observed and displaying membrane and cytosolic localization in HaCaT keratinocytes after 24-h incubation with vehicle (control), PAR2 agonist SLIGKV-NH2 (100 mM) (**a**). Representative images displaying nuclear localization of p65 (*green*) and DAPI (*blue*) in HaCaT keratinocytes after 24-h incubation with vehicle, SLIGKV-NH2 (100 mM) (**c**). Western blot of HaCaT cells following treatment with vehicle (control), SLIGKV-NH2 (100 mM). Samples were probed with antibodies against B1R and tubulin (**b**). B1R mRNA levels were also significantly augmented (**d**). Pretreatment with the NF-κB inhibitor PDTC (25 μM) prevented SLIGKV-NH2-induced B1R expression (**b**, **d**). Values represent mean ± SEM; *P < 0.05; **P < 0.01 by the Student *t* test

To study the effects of the PAR2 agonist, HaCaT keratinocytes were cultured with SLIGKV-NH2 (100 μM) for approximately 24 h. By comparing B1R mRNA and protein levels with the control group, we confirmed that B1R expression was significantly augmented (paired *t* test, *P* < 0.05; Fig. 5b, d). A positive immunocytochemistry reaction for B1R was observed, located on the cell surface in HaCaT keratinocytes cells (Fig. 5a). In addition, SLIGKV-NH2 activated the NF-κB signaling pathway (Fig. 5c), while the NF-κB inhibitor (PDTC: 25 μM) intervened in the regulatory effects of PAR2 introducing B1R expression in HaCaT cells, following B1R down regulation (paired *t* test, *P* < 0.05; Fig. 5b, d).

To understand B1R function on keratinocytes, we assessed whether the B1R antagonist could exert a suppressing action on differentiation or proliferation of HaCaT cell lines. However, these outcomes appeared to occur independently of the epidermal barrier protein filaggrin, since its levels were not affected (the results were not shown). What is more, analysis of cell proliferation of HaCaT cells show that B1R antagonist R892 does not inhibit HaCaT cell growth by pretreatment of PAR2 agonist (the results were not shown).

## Discussion

Following the study of pain, we have spent much effort on the mechanism of acute and chronic itching in recent years, but more work is still required to explore the mechanisms and potential therapy of chronic itching.

We successfully built the AD animal model using DCP-treated mice. Our present study showed that repeated DCP treatment resulted in spontaneous and intolerable itch-like scratching behaviors. The antagonist of B1 kinin receptors significantly inhibited scratching behavior in DCP-treated animals, while the B2R selective antagonist did not. We further confirmed that B1R mRNA and protein were elevated in skin tissues and clearly up regulated in DCP-treated mice compared with the vehicle-treated group. Moreover, a higher B1R staining signal was associated with keratinocyte hyperplasia in this model. Combining B1R expression when superimposed on a PAR2 background in a HaCaT cell (a keratinocytelike surface ectoderm-lineage cell line that naturally expresses PAR1 and PAR2) with our findings, we have reason to believe that B1R contributes to the itching sensation.

It has been reported that B1R is up regulated in various inflammatory states of the animal model [21–24]. In our recent study, the antagonist of B1 kinin receptors significantly inhibited scratching in DCP-treated animals, while the B2R selective antagonist did not. We also observed a marked increase in B1R mRNA and protein levels in the mouse skin tissue 10 days after DCP treatment.

There is an increase in B1R protein expression in DRG neurons 1 day after CFA treatment [25]. Robust calcium transients are observed in the presence of the BK in the primary DRG culture. We sought to find out whether B1R expressed in the neural endings participated directly in the itching sensation. Next, we performed double staining for B1R and the known neuronal marker PGP9.5. Confocal microscopic analysis showed that DCP treatment induced up regulation of B1R expression on the KC cell, while it was delocalized with known neuronal maker PGP9.5 in the skin of DCP-treated mice. So it may function through other mechanisms in the peripheral innervations area of this animal model.

Several reports show that activation of G-protein coupled B2R/B1R correlate with various cell functions [26–29]. It has been reported that B1R is up regulated in various inflammatory states, some of which are mediated by cytokines such as IL-1 [30]. There are also data suggesting that kinin B1R is up regulated by the oxidative stress in the brain of insulin-resistant rats, and its activation causes stereotypic nocifensive behavior through the release of substance P, glutamate, and nitric monoxide (NO) [21].

Increased B1R gene and protein expression in DCP-treated mice provides a structural basis for the important role of BK in chronic inflammation. Here we confirmed that B1R was up regulated in keratinocytes in the skin of DCP-treated mice. Others have reported that keratinocytes produce and secrete various biologically active chemokines, cytokines, and trophic factors to regulate the expression and function of many ion channels in sensory neurons to promote hyperexcitability of sensory terminals [25, 31, 32].

It is thus suggested that the increase of B1R expression facilitates nocifensive behavior through the functional changes in the keratinocytes, which are related by changes in the neuron-keratinocyte cross-talk in the peripheral innervation area.

We postulated that, if human AD resembled that of the rat, many of the same findings would occur in human keratinocyte as in experimental mice treated with DCP. PAR2 is a G-protein-coupled receptor (GPCR) and can be activated in response to various exogenous and endogenous proteases. We extended the findings and enhanced them by characterizing the cellular localization, protein, and mRNA of B1R after pretreatment with PAR2 agonist. In the present study, B1R expression was significantly augmented when pretreated with SLIGKV-NH2 in HaCaT cells; this demonstration is in agreement with our findings in mice.

Several studies show that PAR2/MAPKs/NF-κB signal transduction pathways are involved in PAR2 function [33–35]. The NF-κB family not only plays a pivotal role in inflammation, but also regulates various inflammation factors. In view of the above results, we presented direct evidence that the PAR2 agonist promotes the expression of B1R in keratinocytes. To understand how this was accomplished, we explored the B1R expression in pyrrolidine dithiocarbamate (PDTC) treatment. SLIGKV-NH2 activated the NF-κB signaling pathway, and at the same time under PDTC treatment, B1R expression was decreased because of the blocking of the NF-κB signal transduction pathways.

Searching further for related mechanisms, we found that NF-κB signal pathway activity has an important role in this process. Where PAR2 activation promoted B1R expression, NF-κB inhibitor PDTC decreased B1R expression when pretreatment with SLIGKV-NH2. Under these circumstances, the effects of PAR2 may rely on the NF-κB signal pathway.

Considering the present findings and the above-mentioned reports, we believe that the NF-κB signal pathway has an important role in B1R expression in keratinocytes.

In view of the above results, we assumed that B1R up regulation exerted an important role in the itching sensation in chronic inflammation. To understand how this is accomplished, we explored the role of B1R in differentiating or proliferating HaCaT cell lines. In the present study, analysis of cell proliferation and the gene expression profile of epidermal differentiation markers of HaCaT cells show that B1R antagonist R892 cannot inhibit HaCaT cell growth and proliferation by the pretreatment of PAR2 agonist (results not shown). It has been shown that Klk8 is involved in the differentiation of keratinocytes through up regulation and activation of PAR2 in the late stages of wound healing and proliferation in the early stages [1]. There are also studies on B1R function in the expression of cytokines or signal molecules, such as PGE2 and NO [36–39]. So, different stages in the pathologic and physiologic changes in chronic inflammation may relate to B1R function, which needs further study.

Elevation of kallikrein and BK levels has been well demonstrated in chronic inflammatory conditions. Similarly, we showed how B1R mRNA and protein expression was up regulated in the skin of an experimental animal model. In allergic contact dermatitis with inflammation, B1R may promote scratching as follows: (1) B1R is up regulated in keratinocytes, (2) keratinocytes can also be seen as a “sensory forefront” of neuronal activation and signaling, (3) subsequently, active B1R in the keratinocytes enhances neural sensitization to indirectly promote chronic itching sensations.

In conclusion, the present results indicate that B1R facilitates the chronic itching sensation related to keratinocytes in a DCP-treated chronic inflammation experimental model. How exactly this increase of B1R contributes to chronic itch should be the subject of future investigations.

## Methods

### Animal samples

Male C57BL/6J mice (20–22 g) were used in this study. The dorsal hair of the mice was removed and then stimulated for the first day and the seventh day with DCP repeatedly under conventional conditions. Animal samples comprising skin tissues were obtained from experimental models of chronic inflammation induced by DCP and vehicle-treated mice at the animal center of South-Medical University or Sun Yat-Sen University Cancer Center, Guangzhou, China. The experimental procedures and animal use and care protocols were approved by the Committee on Ethical Use of Animals at the Guangdong Academy of Medical Sciences (Guangzhou, China), following the National Institutes of Health’s animal use and care guidelines.

The reagents used in vivo experiment including: DCP was purchased from Aldrich Chemical Co (Milwaukee, WI, USA). The selective B1R antagonist [Ac-Lys-Arg-Pro-Pro-Gly-(αMe)Phe-Ser-DβNal-IIe, R892] were purchased from Tocris Bioscience (Ellisville, MI, USA). Kallikrein inhibitor NFM, the selective B2R antagonist (Arg-Arg-Pro-Hyp-Gly-Thi-Ser-Tic-Oic-Arg, Hoe140), were obtained from Sigma Chemical Co. (Saint Louis, MO, USA). All chemicals were dissolved according to the manufacturer’s instructions for stock solutions and stored at −20 °C. These chemicals were diluted with NS or miliQ H_2_O to obtain a working solution just before use.

### Immunohistochemistry and immunocytochemistry

We examined skin tissues from experimental models of chronic inflammation induced by DCP including five paired specimens. Immunohistochemical (IHC) staining of those tissues was analyzed. Paraffin-embedded and formalin-fixed samples were cut into 4-μm sections, IHC staining was performed after dewaxing, 10-min incubation was done with 3 % H_2_O_2_ to block endogenous peroxidase, and 25-min microwave antigen was retrieved with B1R antibody (1:500; Biorbyt Ltd, Cambridge, UK) at 4 °C overnight. This was followed by horseradish peroxidase (HRP)-labeled secondary antibody incubation and diaminobenzidine chromogenic reaction in a GT Vision III Detection System/Mo Rb (GeneTech, Shanghai, China). Thereafter, the tissue sections were scanned using multiple spectral microscopy.

For immunocytochemistry analysis, the HaCaT keratinocytes were fixed, permeablized, and then incubated with an ant-NF-κB p65 antibody (1: 400; Cell Signaling, MA, USA), followed by an Alexa Fluor 488-conjugated anti-rabbit IgG (Molecular Probes, Eugene, OR, USA). Paraffin-embedded samples were processed for immunofluorescence as previously described for immunohistochemistry, the sections were incubated with an antibody against B1R antibody (1: 500; Biorbyt Ltd, Cambridge, UK) and PGP9.5 (1:500; Abcam, Cambridge, Mass, USA) for double staining; this was followed by an Alexa Fluor 488-conjugated anti-rabbit IgG (Molecular Probes) and Alexa Fluor 546-conjugated anti-mouse IgG (Molecular Probes). Nuclei were stained with 4′,6-diamidino-2-phenylindole (DAPI). Slides were mounted with antifade mounting medium, dried for 24 h at room temperature, and then stored at 4 °C for future use. Images were captured and analyzed using an Olympus FV1000 confocal laser scanning microscope (Olympus America, Center Valley, PA, USA).

### Western blot

Protein of skin tissues of mice was extracted with ProteoJET Mammalian Cell Lysis Reagent (KeyGen Biotech Co, Nanjing, China) supplemented with protease inhibitor for 10 min on ice. The human HaCaT keratinocytes were washed three times with phosphate buffered saline (PBS), then the pellets were resuspended in lysis buffer supplemented with protease inhibitor for 10 min on ice. After centrifugation at 10,000×*g* for 15 min, the supernatants were dissolved in Loading Buffer and heated at 96 °C for 10 min, resolved on 8 % Tris–HCl polyacrylamide gels, and transferred to a nitrocellulose membrane. The membranes were blocked with 5 % BSA. Overnight incubation (4 °C) of the primary antibody was followed by HRP-conjugated anti-rabbit (1:5000; Novus Biologicals, Littleton, CO, USA) or anti-mouse antibody (1:1000; Novus Biologicals) and Pierce ECL Western Blotting Substrate (Thermo Fisher Scientific, Bothell, WA, USA). Antibody dilution was as follows: B1R (1:500, Santa Cruz, CA, USA); β-tublin (1:1000, Cell Signaling, MA, USA).

### Quantitative real-time PCR

The skin tissues were treated with RNAlater, (Life Technologies, Carlsbad, CA, USA) followed by liquid nitrogen refrigeration were collected. The cultured cells were digested by 0.25 % trypsin-ethylene diaminetetraacetic acid (Life Technologies) and washed twice with PBS before homogenizing. Total RNA was extracted using TRIzol (Life Technologies) according to the manufacturer’s instructions. Relative BDKRB1/Bdkrb1 mRNA expression levels were analyzed by qRT-PCR with double-stranded, DNA-binding dyes as reporters, using GoTaq qPCR Master Mix (Promega, Madison, WI, USA). Glyceraldehyde-3-phosphate dehydrogenase (GAPDH) mRNA expression was used as the internal reference. RT-PCR primers were: Bdkrb1 forward: 5’-CCCAACTACAGTTGTGAACGC-3’, Bdkrb1reverse: 5’-AGGATGATGCCATGCACAGT-3’; Gapdh forward: 5’-CTCCTCCTGTTCGACAGTCAGC-3’; Gapdh reverse: 5’-CCCAATACGACCAAATCCGTT-3’; BDKRB1 forward: 5’-GGCAGCTTCTGATCTGGTGT-3’, BDKRB1 reverse: 5’-GTAGCGGTCCTGACTGATGG-3’; GAPDH forward: 5’-GTGTTCCTACCCCCAATGTGT-3’; GAPDH reverse: 5’-TGAAGTCGCAGGAGACAACC-3’.

### Cell lines

The human HaCaT keratinocytes were cultured in Dulbecco’s modified Eagle’s medium (Life Technologies) supplemented with 10 % fetal bovine serum (FBS; Life Technologies). Cell lines were incubated at 37 °C with 5 % CO_2_ and 95 % humidity. All cells were obtained from Cell Bank, Shanghai Institutes for Biological Sciences (Shanghai, China).

The reagents used in vitro experiment including: PAR2 agonist, Ser-Leu-lle-Gly-Lys-Val-NH2 (SLIGKV-NH2) were purchased from Tocris Bioscience (Ellisville, MI, USA); NF-κB inhibitor pyrrolidine dithiocarbamate (PDTC) was obtained from Sigma Chemical Co. (Saint Louis, MO, USA). All chemicals were dissolved according to the manufacturer’s instructions for stock solutions and stored at −20 °C. These chemicals were diluted with miliQ H_2_O to obtain a working solution just before use.

### Statistical analysis

Data are shown as mean ± SEM. The statistical significance of differences between groups was determined by Student’s *t* test. All data were analyzed using two-tailed tests unless otherwise specified (*P < 0.05; **P < 0.01; ***P < 0.001).
